# The antibacterial activity, antioxidant activity and selectivity index of leaf extracts of thirteen South African tree species used in ethnoveterinary medicine to treat helminth infections

**DOI:** 10.1186/1746-6148-10-52

**Published:** 2014-03-03

**Authors:** Mathew Adamu, Vinny Naidoo, Jacobus N Eloff

**Affiliations:** 1 Phytomedicine Programme, Department of Paraclinical Sciences, Faculty of Veterinary Science, University of Pretoria, Private Bag X04, Onderstepoort, Pretoria 0110, South Africa; 2 Permanent Address: Department of Veterinary Parasitology and Entomology College of Veterinary Medicine, University of Agriculture Makurdi, Makurdi, Nigeria

## Abstract

**Background:**

Diseases caused by bacteria remain a major challenge globally and particularly in sub-Saharan Africa. The plants used in this study have been used in South Africa to treat helminth infections in livestock and humans. In a previous study we found a correlation between antifungal and anthelmintic activity in some cases. In this study we examined other potential uses of these thirteen plant species by determining the antibacterial and antioxidant activity of the leaf acetone extracts.

The antibacterial activity was determined by using a serial microdilution method against *Staphylococcus aureus, Pseudomonas aeruginosa, Escherichia coli* and *Enterococcus faecalis*. Bioautography was used to determine the number of antibacterial compounds. The antioxidant activity was determined using the ABTS and DPPH methods.

**Results:**

*Maesa lanceolata* and *Leucosidea sericea* with an MIC of 0.02 mg/ml had excellent antibacterial activity against *Enterococcus faecalis* and *Pseudomonas aeruginosa.* There was a poor correlation between antioxidant activity and antibacterial activity with R^2^ = 0.143. This is because antibacterial activity is mainly related to non-polar compounds and antioxidant activity to polar compounds. *Maesa lanceolata* extracts had a low cytotoxicity with a selectivity index of 5.2, 2.6, 2.6 and 1.3 for *P. aeruginosa, E. faecalis, E. coli* and *S. aureus* respectively. *Strychnos mitis* extracts had a therapeutic index of 1.1 for *E. coli*.

**Conclusions:**

This study shows that plant extracts of some species used in ethnoveterinary medicine as anthelmintic may also have excellent antibacterial activity.

## Background

Resistance to available antibiotics is increasing at a very alarming stage globally [1]. Efforts are urgently needed to replace current available antibiotics. This resistance is complex especially in sub-Saharan Africa due to the incidence of HIV/AIDS and diseases such as tuberculosis decreasing the immune activity of hosts. The situation is complicated by poor sanitary conditions and poor access to potable water mainly due to poverty. If antibiotics are available they are too expensive for the poorer communities. In some cases especially in the rural areas antibiotics are adulterated and of little value in the treatment of diseases caused by bacteria to humans and animals [2]. The antibacterial activity of plants is continuously attracting global attention [3,4]. Parasitic infection may lead to the release of free radicals which may have severe consequences on cellular metabolism. The anthelmintic activity may therefore be due to the presence of antioxidants in extracts that have the potential to prevent the activity of free radicals and reactive oxygen species thus helping in fighting diseases caused by bacteria and other pathogens.

South Africa with 10% of the vascular plant species diversity of the world is a potential source of undiscovered compounds with high activity of extracts against a variety of bacterial and fungal pathogens [5]. The plant species used in this study were chosen based on evidence of traditional use as anthelmintics and antibacterial as well as availability. This investigation will assist in identifying plants for further study aimed at identifying compounds as potential source of new antibiotic for primary health care needs of man and his animals. If plant extracts that have anthelmintic activity are also useful in combating bacterial pathogens it could have a double value.

These plants were originally investigated for its activity against *Haemonchus contortus* a very important helminth of sheep. Some of the extracts had promising activity [6]. There were also promising activity against certain important fungal pathogens [7]. Some of these extracts had a potential to be used to treat animal diseases caused by fungi or helminths. It will be interesting to determine the potential use against bacteria and also the degree to which some of these extracts would have antioxidant activity that could address inflammations caused by microbes or helminths. The aim of this study therefore was to determine the antibacterial and antioxidant activity of thirteen selected tree species against two Gram-positive and two Gram-negative bacteria. A secondary aim was to calculate the selectivity index (also known as therapeutic index) of these extracts in combating bacteria. One of the parameters that plays a role in selecting the best plant species for development of a product is the total activity. By dividing the mass in mg extracted from 1 g of plant material with the MIC in mg/ml a value is obtained that indicates to what level the active compounds in 1 g of plant material can be diluted and still retain the activity against the pathogen.

## Methods

### Plant collection

Leaves of thirteen plant species were collected in November 2009 at the Pretoria National Botanical Garden in South Africa. The trees were identified and labeled and voucher specimens were made and stored in the HGW Schweickert Herbarium of the University of Pretoria with voucher numbers (Table 1). The leaves were dried at room temperature in a ventilated room, milled to a fine powder in Macsalab Mill (Model 2000 LAB Eriez^®^) and stored in closed containers in the dark until use.

**Table 1:** List of plant species used in the investigation, their traditional uses and references

**Plant species**	**Family**	**Medicinal uses**	**Reference**
*Brachylaena discolor*	Asteraceae(267)	Purgatives against intestinal parasites, anthelmintics for calves, sheep and goats	[8-10]
*Zanthoxylum capense*	Rutaceae(96)	Gastric and intestinal disorders, anthelmintics, cough, bronchitis, pleurisy	[8,10]
*Clerodendrum glabrum*	Lamiaceae(403)	Intestinal parasites, coughs, fever and diabetes	[8-10]
*Heteromorpha trifoliata*	Apiaceae(491)	Intestinal worms, colic in horses and vermifuge, enemas for abdominal disorders	[8,9,11]
*Apodytes dimidiata*	Icacinaceae(139)	Enemas for intestinal parasites, purgatives, inflammation of the ear	[8,9,11]
*Strychnos mitis*	Strychnaceae(73)	Malaria, fevers	[12]
*Maesa lanceolata*	Maesaceae(615)	Anthelmintics, treatment of wounds and infertility	[9]
*Indigofera frutescens*	Papilionaceae(675)	Anthelmintics	[9]
*Leucosidea sericea*	Rosaceae(288)	Treatment of opthalmia, anthelmintics, astringents and vermifuge	[9,13]
*Melia azedarach*	Meliaceae(702)	Effective anthelmintics, emetic, cathartic and treatment of eczema	[9,10,14]
*Clausena anisata*	Rutaceae(317)	Anthelmintics, purgatives, rheumatism, fevers and myiasis	[15]
*Cyathea dregei*	Cyatheaceae(658)	Anthelmintics	[15]
*Millettia grandis*	Papilionaceae(704)	Anthelmintics and tranquilizers	[9,16]

Only the leaves of these plants species were used in the study. Figures in brackets after family name are PRU voucher specimen numbers. (Table from Adamu et al., [6]).

### Plant extraction

Plant material from each species investigated was extracted with acetone, (technical grade, Merck) in polyester centrifuge tubes at a ratio of 10 ml/g and repeated twice. Acetone was selected based on its superiority as extractant based on a number of parameters [17] especially because it extracts compounds with a wide range of polarities and has low toxicity to humans and to microorganisms. The tubes were vigorously shaken for 30 min on an orbital shaker. Tubes were centrifuged at 4000 x g for 10 min and the supernatant was filtered using Whatman No.1 filter paper before being transferred into pre-weighed glass containers. Acetone was evaporated under a stream of air in a fume hood at room temperature to produce the dried extract and for quantification [18].

### Chromatographic analysis

The extracted chemical components were analysed by separation with thin layer chromatography (TLC) using aluminium-backed TLC plates (Merck, Silica gel F254). The TLC plates were developed in saturated chambers using mobile phases of varying polarities, namely, ethyl acetate/methanol/water (40:5.4:5) [EMW] (polar/neutral), chloroform/ethyl acetate/formic acid (5:4:1) [CEF] (intermediate polarity/acidic) and benzene/ethanol/ammonia hydroxide (90:10:1) [BEA] (non-polar/basic) [19]. Separated compounds were visualised under UV light at wavelengths of 254 and 365 nm after which TLC plates were sprayed with vanillin-sulphuric acid and heated at 110°C for optimal colour development [20].

### Antioxidant activity

Antioxidant activity was determined by using qualitative and quantitative methods. For the qualitative antioxidant activity TLC chromatograms of extracts were sprayed with 0.2% 1, 1-diphenyl-2-picryl-hydrazyl (DPPH) (Sigma) in methanol as an indicator [21]. The quantitative antioxidant activities were determined using the ABTS (2,2′-azinobis-(3-ethylbenzothiazoline-6-sulfonic acid) and DPPH methods [22,23]. These methods compare the antioxidant activity of the extracts to that of the standards ABTS and DPPH by measuring the decolorization of extracts spectrophotometrically. The EC_50_ values for the DPPH analysis was determined by plotting activity against concentration and determining which concentration would have yielded an activity of 50%.

### Antibacterial activity

The MIC values were determined using a serial microplate dilution method [24]. Bacterial organisms used were *Staphylococcus aureus* (ATCC 29213), *Enterococcus faecalis* (ATCC 29212), *Pseudomonas aeruginosa* (ATCC 27853) and *Escherichia coli* (ATCC 25922). These strains are recommended for antibacterial activity testing by the United States National Committee for clinical laboratory standards [25]. Bacterial cells were inoculated into fresh Müller–Hinton (MH) broth (Fluka, Switzerland) and incubated at 37°C overnight and density determined prior to the screening procedures. Densities of bacterial cultures after incubation overnight were diluted to the following:: *Staphylococcus aureus*, 2.6 × 10^12^ cfu/ml; *Enterococcus faecalis*, 1.5 × 10^10^ cfu/ml; *Pseudomonas aeruginosa*, 5.2 × 10^13^ cfu/ml; *Escherichia coli*, 3.0 × 10^11^ cfu/ml. One of the key aspects are that a 50% inoculum was used, this led to enhanced growth with no lag phase and eliminated possible complications due to infections by spores of other microorganisms [24]. Acetone was used as solvent control, while gentamicin was the positive drug control.

### Bioautographic investigations

Bioautography is a specialized form of planar chromatography where the separated compounds are not made visible by treating with a spray reagent but are sprayed with a live microorganism, incubated and then sprayed with a chemical that indicates whether the microorganism grew or not. This makes it possible to determine how many different antimicrobial compounds were separated and also to calculate the R_f_ value of the bioactive compounds. Thin layer chromatography (TLC) plates were loaded with 10 μl of 10 mg/ml of extract and dried before developing in mobile phases of BEA, CEF and EMW. The solvents were completely evaporated from the plates in a stream of air. Plates were then sprayed with concentrated cultures of bacteria until completely moist using a spraying gun coupled to an air pump. The moist plates were incubated overnight at 37°C in a sealed container in an incubator under 100% relative humidity. The plates were then sprayed with 2 mg/ml of *p-*iodonitrotetrazolium violet (INT) (Sigma) and incubated for a further 2 h or until clear growth was apparent [26]. The emergence of purple-red colour resulting from the reduction of INT into its respective formazan was a positive indicator of cell viability [26]. Clear zones against the purple background indicated antibacterial activity of compounds separated on the chromatogram.

## Results

### Plant extracts yield

Leaves of the thirteen plant species were extracted using acetone as the extracting solvent, this gave different percentage yields(Table 2), with *Leucosidea sericea* having the highest yield of 6.3%, (6.3 mg extracted out of every 100 mg dried leaf material) followed closely by *Apodytes dimidiata* with 6.1%. The lowest yield was obtained with *Zanthoxylum capense* (0.8%).

**Table 2:** Percentage yield and antioxidant activity of thirteen South African plant extracts used for antibacterial activity

**Plant species**	**% Yield**	**EC**_**50 **_**DPPH**	**TEAC**
*Heteromorpha trifoliata*	3.3	4.36	0.2
*Indigofera frutescens*	0.8	0	0.5
*Zanthoxylum capense*	1.6	4.0	0.4
*Milletia grandis*	1.3	4.6	0.6
*Brachylaena discolor*	6.1	2.6	0.2
*Clerodendrum glabrum*	3.8	3.5	0.5
*Strychnos mitis*	2.8	3.5	0.3
*Cyathea dregei*	2.1	3	0.4
*Apodytes dimidiata*	6.3	3.5	0.3
*Melia azedarach*	2.3	3.3	0.8
*Clausena anisata*	3.4	2.5	0.2
*Maesa lanceolata*	2.5	1.4	1.2
*Leucosidea sericea*	1.2	0.0	0.7

EC_50_ concentration that leads to 50% reduction in DPPH activity (the lower the value the higher the activity), TEAC Trolox equivalent antioxidant assay. Activity compared to trolox (the higher the value the higher the activity).

### Phytochemical profiling and antioxidant activity

**Figure 1:**
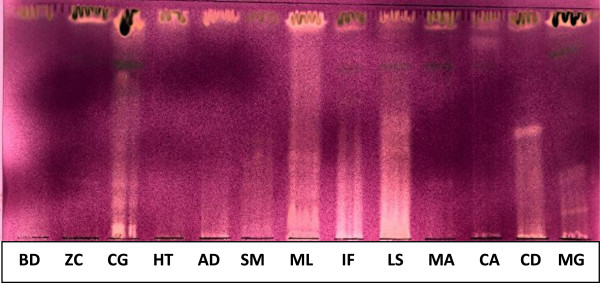
**Chromatogram developed with EMW and sprayed with DPPH.** Yellow bands indicate separated compounds with antioxidant activity KEY: BD, *Brachylaena discolor*; ZC, *Zanthoxylum capense*; CG, *Clerodendrum glabrum*; HT, *Heteromorpha trifoliata*; AD, *Apodytes dimidiata*; SM, *Strychnos mitis*; ML, *Maesa lanceolata*; IF, *Indigofera frutescens*; LS, *Leucosidea sericea*; MA, *Melia azedarach*; CA, *Clausena anisata*; CD, *Cyathea dregei*; MG, *Milletia grandis*.

Extracts of plants contain many chemical compounds (7). The compounds varied from polar to non-polar and in some extracts compounds of intermediate polarity were observed (Figure 1). *Maesa lanceolata* (ML) had the most antioxidant compounds (Figure 1) followed by *L. sericea* (LS). This pattern was consistent in all 3 solvent systems (BEA, EMW and CEF) used and was supported by the ABTS and DPPH assays used for quantification of the antioxidant activity (Table 2). *M. lanceolata* with a TEAC of 1.20 and *L. sericea* with an EC_50_ of 0.0051 had the best antioxidant activity using ABTS and DPPH respectively.

### Bioautography

**Figure 2:**
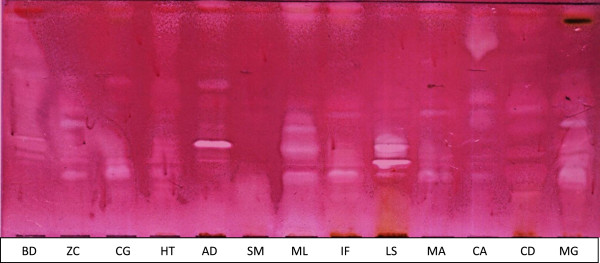
**Bioautogram developed with BEA of different plant leaf acetone extracts against *E. coli *showing antibacterial bands**. White bands indicate separated compounds that inhibited the growth of *E. coli* KEY. BD, *Brachylaena discolor*; ZC, *Zanthoxylum capense*; CG, *Clerodendrum glabrum*; HT, *Heteromorpha trifoliata*; AD, *Apodytes dimidiata*; SM, *Strychnos mitis*; ML, *Maesa lanceolata*; IF, *Indigofera frutescens*; LS, *Leucosidea sericea*; MA, *Melia azedarach*; CA, *Clausena anisata*; CD, *Cyathea dregei*; MG, *Milletia grandis*.

Three solvent systems were used. The non-polar BEA separated 56% of the total active bands against the four bacteria indicating that the antibacterial compounds were non-polar. The position of active compounds on the chromatogram relative to the front (R_f_) should be consistent under similar conditions and is calculated by dividing the distance the compound travelled with the distance of the front of the eluent. The highest number of active bands was in the *L. sericea* extract separated with BEA, (Figure 2) with 6 active bands against *E. coli* (R_f_ values; 2.35, 2.67, 2.86, 3.20, 3.48 and 4.0) and *M. lanceolata* with 4 active bands (R_f_ values; 1.07, 2.29, 2.67 and 4.00). *S. mitis* had one active band (R_f_ 1.22) in BEA solvent against *S. aureus*. A compound with R_f_ value of 4.00 was active in *Zanthoxylum capense* (2.11, 4.00), *Clerodendrum glabrum* (4.00)*, Heteromorpha trifoliata* (4.0), *Maesa lanceolata, Indigofera frutescens* (2.00, 4.00), *Leucosidea sericea, Melia azedarach* (2.00, 4.00), *Clausena anisata* (1.14, 4.00) and *Milletia grandis (*2.00, 3.48, 4.00)*.* This may mean that the same antibacterial compound is present in extracts of nine species. The 13 plant extracts had more active bands against *E. coli* than other tested organisms. In total, 75 active bands were recorded against the four bacteria.

### Minimal inhibitory concentrations and total activity against bacterial pathogens

The minimal inhibitory concentration (MIC) for the antibacterial activity of the leaf extracts and cytotoxicity of the plant extracts are presented in Tables 3 and 4. *L. sericea and M. lanceolata* extracts with MIC of 0.02 mg/ml against *E. faecalis and P. aeruginosa* were the best for this study. This was followed by 0.04 mg/ml for *M. lanceolata* against *P. aeruginosa* and 0.08 mg/ml for *L. sericea* and *M. lanceolata* against *S. aureus, E. coli* and *S. aureus* respectively. Other plant extracts with good MIC values were *C. anisata, I. frutescens, B. discolor* and *M. azedarach* with *MIC of 0.16* mg/ml*.* The other plant extracts had moderate MIC values with the exception of *Z. capense* and *A. dimidiata* with poor MIC values as high as 2.5 mg/ml. There is some consensus that MIC values of 0.10 mg/ml or less are considered good (18, 24) while values up to 0.32 mg/ml are reasonable. MIC values above 0.64 are considered as having poor activity. *Pseudomonas aeruginosa* and *Enterococcus faecalis* were the most susceptible bacteria organisms among the bacteria tested.

**Table 3:** Minimal inhibitory concentrations (MIC) (lowest concentration that inhibits the growth of the pathogen) in mg/ml of the leaf extracts of 13 plant species evaluated for antibacterial activity using four bacteria organisms

**Plant species**	***S. aureus***	***E. coli***	***E. faecalis***	***P. aeruginosa***
*Brachylaena discolor*	1.25	0.63	0.16	0.31
*Zanthoxylum capense*	1.25	2.50	0.31	0.31
*Clerodendrum glabrum*	1.25	0.31	1.25	0.63
*Heteromorpha trifoliata*	1.25	0.63	1.25	0.63
*Apodytes dimidiata*	0.31	2.50	1.25	0.31
*Strychnos mitis*	0.31	0.04	0.63	0.16
*Maesa lanceolata*	0.08	0.04	0.04	0.02
*Indigofera frutescens*	0.16	0.16	0.08	0.31
*Leucosidea sericea*	0.08	0.08	0.02	0.02
*Melia azedarach*	0.63	0.31	0.16	0.63
*Clausena anisata*	0.16	0.31	0.31	0.31
*Cyathea dregei*	0.31	0.31	1.25	0.31
*Milletia grandis*	0.31	0.31	0.31	0.31
Gentamicin	<0.02	<0.02	<0.02	<0.02
Acetone	>25%	>25%	>25%	>25%

**Table 4:** **Cytotoxicity from Adamu et al., [**[6]**] and selectivity index of leaf acetone extracts of 13 plant species against four bacterial pathogens calculated by dividing cytotoxicity by MIC**

**Plant species**	**Cytotoxicity (mg/ml)**	***Staphylococcus aureus***	***Escherichia coli***	***Enterococcus faecalis***	***Pseudomonas aeruginosa***
***Brachylaena discolor***	0.004	0.00	0.01	0.03	0.01
***Zanthoxylum capense***	0.008	0.01	0.00	0.03	0.03
***Clerodendrum glabrum***	0.172	0.14	0.55	0.14	0.27
***Heteromorpha trifoliata***	0.043	0.03	0.07	0.03	0.07
***Apodytes dimidiata***	0.003	0.01	0.00	0.00	0.01
***Strychnos mitis***	0.043	0.14	1.08	0.07	0.27
***Maesa lanceolata***	0.104	1.30	2.60	2.60	5.20
***Indigofera frutescens***	0.052	0.33	0.33	0.65	0.17
***Leucosidea sericea***	0.016	0.20	0.22	0.80	0.80
***Melia azedarach***	0.145	0.23	0.47	0.91	0.23
***Clausena anisata***	0.053	0.33	0.17	0.17	0.17
***Cyathea dregei***	0.017	0.05	0.05	0.01	0.05
***Milletia grandis***	0.021	0.07	0.07	0.07	0.07

Total activity depends not only on the antibacterial activity of the extract, but also on the quantity extracted from the plant. *L. sericea* had the best total activity of 6265 ml/g against *P. aeruginosa* and *E. faecalis* (Table 5)*.* This means that the extract from one gram of the plant material could be diluted to 6265 ml and still retains activity against the microorganism. The two plants extracts with the lowest total activity value were *Z. capense* and *H. trifoliata* with 6.5 and 13 ml/g respectively.

**Table 5:** Total activity in ml/g of the leaf extracts of 13 plant species screened for antibacterial activity against four bacterial pathogens

**Plant species**	***Staphylococcus aureus***	***Escherichia coli***	***Enterococcus faecalis***	***Pseudomonas aeruginosa***
*Brachylaena discolor*	52.8	104.8	412.5	213
*Zanthoxylum capense*	13	6.5	52.3	52.3
*Clerodendrum glabrum*	25.5	102.9	25.5	50.6
*Heteromorpha trifoliata*	20.4	40.5	20.4	40.5
*Apodytes dimidiata*	391.6	48.6	97.1	391.6
*Strychnos mitis*	241.9	1875	119.1	468.8
*Maesa lanceolata*	697.5	1395	1395.0	2790
*Indigofera frutescens*	256.3	256.3	512.5	132.3
*Leucosidea sericea*	1566.3	1566.3	6265.0	6265
*Melia azedarach*	72.7	147.7	286.3	72.7
*Clausena anisata*	424.4	219	219	219
*Cyathea dregei*	161.3	161.3	40	161.3
*Milletia grandis*	79.7	79.7	80	79.7

## Discussion

In general extracts with a good antioxidant activity (low EC_50_ or high TEAC) had a low antibacterial (high MIC) activity. The correlation coefficient between MIC and DPPH was 0.0118 and between MIC and TEAC was 0.0068. We have frequently found that nonpolar extracts or compounds from plant extracts had high antibacterial activity and polar compounds had low antibacterial activity. In the case of *L. sericea* and *M. lanceolata* we found good antioxidant activity with TEAC values of 0.7 and 1.2 and good antimicrobial activity (MIC 0.04 mg/ml for all four bacteria). It is possible that these extracts may contain tannins that are known to have good antioxidant and antibacterial activity. The results show that plants extracts are active against both Gram positive and Gram negative bacteria. It has frequently been stated that plant extracts are more active against Gram positive bacteria than against Gram negative bacteria [27]. This may be due to the cell walls of Gram negative bacteria being less permeable to antimicrobial compounds [28]. With the plant extracts used here the average antibacterial activity against the Gram negative bacteria was slightly better than the activity against Gram positive bacteria (MIC of 0.48 vs. 0.55 mg/ml).

Based on the selectivity index, that is the ratio of MIC to cytotoxicity value, the *M. lanceolata* extract had the best selectivity index of 5.2. The results make such plant extracts good candidates for further study. The selectivity value helps to differentiate activity that is due to general toxicity and one that is selectively toxic to microorganisms.

Extracts of *L. sericea* and *M. lanceolata* had the best antibacterial activity with an MIC of 0.02 mg/ml in this study. Previous study by Bosman et al., [29] reported that *L. sericea* had activity against bacteria organisms using the disc diffusion method, but no activity was reported for acetone leaf extracts of *L. sericea.* This study does not agree with their findings. This is probably due to the different methods used. The microdilution method we used is more sensitive than the disc diffusion method [24]. Recently, Aremu et al., [30] reported an MIC of 0.025 mg/ml using petroleum ether and dichloromethane leaf extracts of *L. sericea* against *Bacillus subtilis* and *Staphylococcus aureus*. The solvent used in our study differed to that used in their study. With solvent known to extract compounds of different polarity from same plant, this may explain the different MIC values of 0.04 mg/ml for S*. aureus* as compared to 0.025 mg/ml by Aremu et al., [30]. The activity of *L. sericea* may be due to the presence of active compounds such as aspindinol [29], alkaloids, phenolics and saponins [30] that had previously been reported from this plant.

*Maesa lanceolata* had an MIC of 0.02 mg/ml, this is the first report of such a good activity from this plant extract. Previous study by Sindambiwe et al., [31] reported that the plant extract did not have antibacterial activity even at high concentration of 10 mg/ml. The results differ probably due to the method in use, as they used the agar dilution and broth dilution method for antibacterial and antifungal assays respectively. The poor antibacterial activity shown by *Z. capense* in this study agrees with past study by McGaw et al., [32].

The results of the bioautography correlates positively with the MIC results, for example *L. sericea* had 6 compounds in the BEA system, while *M. lanceolata* had 4 compounds and a corresponding MIC value of 0.04 mg/ml as the best average MIC value for all four bacteria tested. Previously, report on the anthelmintic [6] and antifungal activity [7] of acetone extracts of these plant species have shown varied degree of activity. The most promising plant extracts against egg hatching and larval development of *Haemonchus contortus* were *H. trifoliata, M. lanceolata* and *L. sericea*. The most promising extracts based on activity against fungal pathogens were *Clausena anisata, Clerodendrum glabrum, Milletia grandis* and *Zanthoxylum capense*. The activity of *L. sericea* and *M. lanceolata* against bacterial pathogens in this study was the best. Based on the activity of these plant extracts against the eggs and larvae of *H. contortus*, bacteria, fungi and their cytotoxicity. *Leucosidea sericea* was selected for further study which involves activity guided fractionation and isolation of compounds of the most promising plants extracts and testing activity against egg and larvae of *Haemonchus contortus,* toxicity against Vero cell lines.

## Conclusions

Some leaf extracts of plants used for anthelmintic purposes have good antibacterial activity or good antioxidant activity. It is possible that extracts with a good anthelmintic and antimicrobial activity may be active against a general metabolic system. If it had an effect on the Krebs cycle or electron transport chain, these extracts would also be toxic against animal cells. The selectivity index provides an indication of the safety of the extract. In the case of *L. sericea* and *M. lanceolata* the acetone extracts had promising activity, promising safety and with a total activity of higher than 1 litre/g also a very promising yield from tree leaves. In most cases the antioxidant activity was relatively low, This may mean that the effect on helminths is due to an effect on the worms and not on a general increase in the immune system of the host.

## Competing interests

The authors declare that they have no competing interests.

## Authors’ contributions

MA participated in the design of the study, carried out field work, prepared the extracts, participated in all assays and wrote the first draft of the manuscript. VN participated in the design and coordination of the study, supervised the study and revised the draft manuscript. JNE conceived the study, participated in the design and coordination of the study, supervised the study, analysed the data and revised the final manuscript. All authors read and approved the final manuscript.
